# Thin Film Silicon Nanowire/PEDOT:PSS Hybrid Solar Cells with Surface Treatment

**DOI:** 10.1186/s11671-016-1527-1

**Published:** 2016-06-29

**Authors:** Hao Wang, Jianxiong Wang, Lei Hong, Yew Heng Tan, Chuan Seng Tan, Rusli

**Affiliations:** NOVITAS, Nanoelectronics Centre of Excellence, School of Electrical and Electronic Engineering, Nanyang Technological University, 50 Nanyang Avenue, Singapore, 639798 Singapore

**Keywords:** Hybrid solar cell, Si nanowires, PEDOT:PSS, Surface treatment

## Abstract

SiNW/PEDOT:PSS hybrid solar cells are fabricated on 10.6-μm-thick crystalline Si thin films. Cells with Si nanowires (SiNWs) of different lengths fabricated using the metal-catalyzed electroless etching (MCEE) technique have been investigated. A surface treatment process using oxygen plasma has been applied to improve the surface quality of the SiNWs, and the optimized cell with 0.7-μm-long SiNWs achieved a power conversion efficiency (PCE) of 7.83 %. The surface treatment process is found to remove surface defects and passivate the SiNWs and substantially improve the average open circuit voltage from 0.461 to 0.562 V for the optimized cell. The light harvesting capability of the SiNWs has also been investigated theoretically using optical simulation. It is found that the inherent randomness of the MCEE SiNWs, in terms of their diameter and spacing, accounts for the excellent light harvesting capability. In comparison, periodic SiNWs of comparable dimensions have been shown to exhibit much poorer trapping and absorption of light.

## Background

In recent years, extensive research has been devoted towards rendering solar energy more cost competitive to be a viable energy source. For example, Si nanostructures, such as Si nanowires (SiNWs) have been incorporated into solar cells for light trapping, so that thinner Si absorber layer can be used to lower the material cost [1–3]. SiNWs fabricated by the low cost solution-based metal-catalyzed electroless etching (MCEE) technique have also been combined with organic semiconductors to form hybrid solar cells [4, 5]. Such cells present a very cost-effective option due to their simple structure, coupled with the solution-based, low temperature and large area fabrication process. Currently, a promising power conversion efficiency (PCE) of 13.01 % has been reported for hybrid solar cells based on SiNWs and poly(3,4-ethylene-dioxythiophene):polystyrenesulfonate (PEDOT:PSS) [6]. A high PCE of 17.4 % [7] has also been reported for bulk Si/PEDOT:PSS cell based on a backPEDOT cell structure, where light is incident on Si instead of PEDOT:PSS to reduce parasitic absorption loss in the PEDOT:PSS. Despite the outstanding efficiency achieved, it should be noted that the hybrid cells demonstrated using expensive bulk Si wafer are not a practical option for low cost applications. It is imperative that lower cost Si thin films should be explored instead. We have previously reported SiNW/PEDOT:PSS hybrid solar cells based on a 2.2-μm Si thin film that achieved a PCE of 5.6 % [8]. The performance of the SiNW-based cells was noted to be only marginally improved compared to their planar counterpart with the same thickness of Si thin film. This has been attributed to the high recombination rate associated with the defective surface of the SiNWs prepared by the MCEE technique, as well as the lower shunt resistance of the SiNW-based cells [9, 10]. To further improve the performance of thin film SiNW/PEDOT:PSS solar cells, in this work, we fabricate such cells using a thicker Si thin film of 10.6 μm. To address the high surface recombination associated with the SiNWs, we apply a recently developed two-step surface treatment process to improve the surface quality of the SiNWs [11]. We achieve a PCE of 7.83 % for the treated SiNW/PEDOT:PSS cells with an optimized SiNW length of 0.7 μm. The average *V*_oc_ of the SiNW/PEDOT:PSS cells is improved from 0.461 to 0.562 V as compared to the untreated counterparts. The results in this study demonstrate the potential of thin film Si/PEDOT:PSS hybrid cells incorporated with SiNWs for light trapping, and the importance of surface treatment to fully realize the advantages brought about with the use of SiNWs.

The MCEE approach can readily produce SiNWs with 20–300 nm diameter by simply immersing the Si films into an aqueous HF solution which contains metal catalyst such as metal particles (silver or gold) or metal ions, and oxidizing agents such as Fe(NO_3_) or H_2_O_2_ [12]. HF-AgNO_3_ solution has been widely used for the MCEE SiNW fabrication since 2002, as reported by Peng et al. [13]. The MCEE SiNWs offer excellent light trapping ability as evidenced from their generally low reflectance in the visible light range. The light reflectance can be as low as 1.4 % over the wavelength (*λ*) range of 300–600 nm for SiNWs fabricated on bulk crystalline Si wafers [12]. SiNW array films fabricated on glass substrates by the MCEE technique have also shown low reflectance of less than 10 % from 300 nm < *λ <* 800 nm and strong broadband optical absorption of more than 90 % [14, 15]. AgNO_3_ prepared MCEE SiNW hybrid cells exhibited low reflectance of <5 % over a broad range 300 nm < *λ <* 1050 nm for nanowire length greater than 1 μm [16]. The results are interesting given that the dimensions of the MCEE SiNWs commonly etched using AgNO3/HF solution, with diameters from 30–150 nm and spacing of 20–80 nm [4], are not in the optimized range for effective scattering of the main solar spectrum. This leads to the question of the origin of their strong light trapping properties. Recently, interest in the effect of disorders on the optical performance of Si nanostructures has grown [17–20]. Some theoretical studies have been done on the SiNW arrays without any underlying Si thin film using the finite-difference time-domain (FDTD) method [17, 18] and transfer matrix method (TMM) [19]. In these studies, individual structural parameters of the SiNW arrays such as diameter [18], position [17–19], and length [18] have been varied one at a time to study their effects on the optical properties of the SiNW arrays. The results have shown that the disorders in the SiNW arrays resulted in improved light absorption as compared to the periodic structure with comparable dimensions, attributed to the presence of additional resonance modes and broadening of the existing modes [17–19]. In this work, we have carried out optical simulations based on a hybrid structure of random SiNW arrays on an underlying Si thin film, and with PEDOT:PSS on top using the finite element method (FEM) [21]. We investigate the effects of the randomness of the MCEE SiNW arrays, in terms of their diameter and spacing, on the optical properties of the hybrid SiNW/PEDOT:PSS solar cells. Instead of varying the parameters randomly one at a time over a pre-defined range, we allow both the SiNW diameter and spacing to vary concurrently, so that the simulated structure is closer to what is observed experimentally. It is found that this has resulted in enhanced scattering and absorption, as compared to the case where the parameters are varied randomly one at a time. Overall, the simulation studies reveal that the inherent randomness of the SiNWs results in a substantial decrease in the reflectance and transmittance of light. Consequently, there is a significant increase in the absorption of light as compared to periodic SiNWs of comparable dimension, but with uniform diameter and spacing. In the presence of SiNW randomness, the ultimate efficiency is boosted from 16.9 to 27.2 % for a simulated hybrid cell based on a 2.2-μm-thick Si absorber, which represents a remarkable 60.6 % improvement.

## Methods

**Fig. 1:**
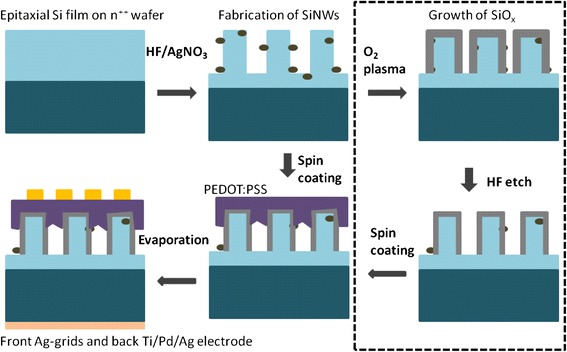
A schematic illustration of the fabrication process for SiNW/PEDOT:PSS hybrid solar cell with surface treatment

**Fig. 2:**
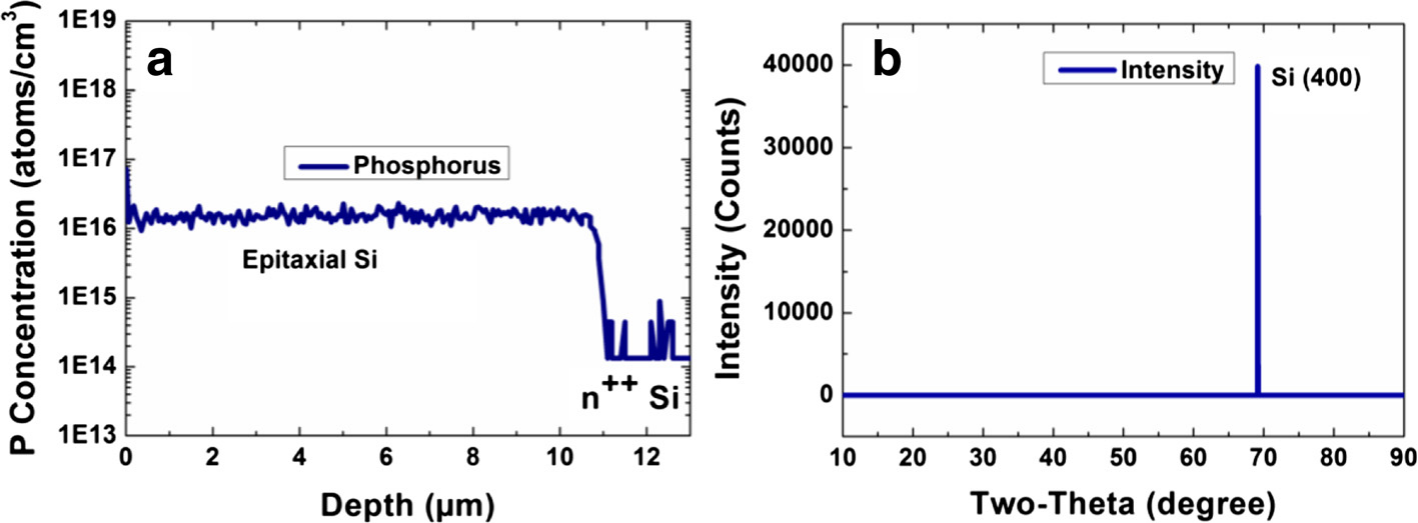
**a** DSIMS profile and **b** XRD profile of the epitaxial Si thin film

Figure 1 illustrates the fabrication process for the SiNW/PEDOT:PSS hybrid solar cells. A crystalline Si layer with a thickness of 10.6 μm and phosphorus doping concentration of 1.5 × 10^16^ cm^−3^ was epitaxially grown on top of n^++^ Si (100) substrate in a rapid thermal chemical vapor deposition (RTCVD) reactor. The n^++^ Si substrate with an arsenic doping concentration of ~1 × 10^20^ cm^−3^ was first cleaned by in situ ultra pure H_2_ at 1100 °C inside the RTCVD reactor, followed by the growth of epitaxial Si layer using dichlorosilane precursor and phosphine (PH_3_) dopant gas at 1000 °C. The doping concentration and thickness of the epitaxial Si layer are confirmed by dynamic secondary ion mass spectrometry (DSIMS) while its crystallinity is verified by X-ray diffraction (XRD) measurements as shown in Fig. 2a, b, respectively. It should be emphasized that as the heavily doped Si substrate has very short minority-carrier diffusion length, optical absorption leads to generation of carriers that recombine readily and do not contribute to photocurrent. Therefore, only the thin Si epilayer is responsible for the generation of photocurrent in the solar cells. Note that the high temperature CVD process used in this work allows us to obtain high quality crystalline Si thin films for the fabrication of SiNW/PEDOT:PSS cells. This enables us to probe the upper limit of the performance of such cells without the complications relating to the quality of the Si thin films. However, the approach is not practical as it is not a low cost process and high temperature is involved. For practical application, other approaches would have to be considered to realize hybrid cells based on Si thin films grown at low temperature and on low cost substrates [22, 23].

SiNWs were fabricated on the epitaxial Si films by the MCEE technique in a solution consisted of 4.6 M HF and 0.02 M silver nitrate (AgNO_3_) [24]. SiNWs with different lengths of *L* = 0.4, 0.7, 0.95, 1.5, and 2.7 μm were fabricated by adjusting the etch time. Following that the top surface of the SiNWs was spin coated with highly conductive PEDOT:PSS (PH1000) mixed with 5 wt% dimethyl sulfoxide (DMSO) at 2800 r/min, and then annealed at 105 °C for 10 min in atmosphere. The cells were completed by depositing electrodes that comprised a layer of Ag grid on the PEDOT:PSS layer and Ti/Pd/Ag on the backside of the Si substrate using e-beam evaporation. Each cell has a size of 0.95 cm^2^ and a 12 % incident light power loss due to the Ag grid shadowing. For the two-step surface treatment process presented in the dotted box in Fig. 1, instead of using ozone [11], we have tried a different approach of oxidizing the surface of SiNWs using oxygen plasma. The SiNWs were first treated in a RF 13.56 MHz inductively coupled oxygen plasma for 480 s to form a layer of sacrificial oxide of ~4–5 nm. The plasma was generated with an O_2_ gas flow of 30 sccm, RF power of 30 W and pressure of 200 mTorr. The SiNWs were then etched in 5 % dilute hydrofluoric (HF) acid for 85 s to partially remove the oxide layer, together with the embedded Ag nanoparticles, leaving behind a thin layer of residual SiO_x_ of ~1 to 2 nm for surface passivation [11]. Our results reveal that the treatment process using oxygen plasma is as effective as the one using ozone which we have reported previously [11].

## Results and Discussion

**Fig. 3:**
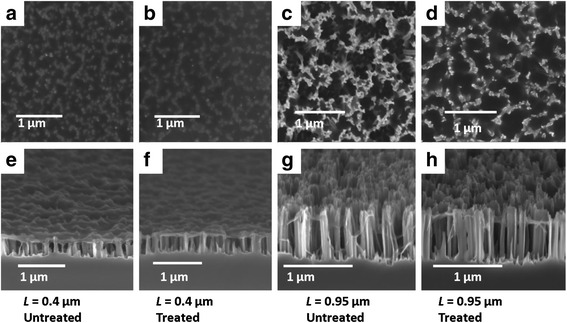
**a**, **b** The top view SEM images of 0.4-μm-long SiNWs/PEDOT:PSS without and with surface treatment, respectively; **c**, **d** the top view SEM images of 0.95-μm-long SiNWs/PEDOT:PSS without and with surface treatment, respectively; **e**–**h** the corresponding cross-sectional view SEM images of these samples

**Fig. 4:**
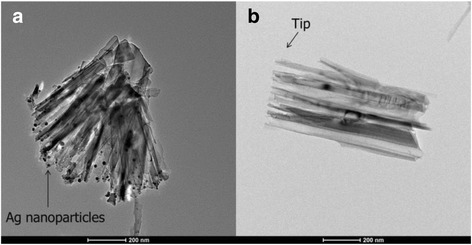
TEM images of the SiNWs **a** before and **b** after the two-step surface treatment

**Fig. 5:**
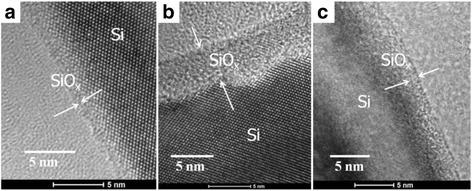
HRTEM images of the SiNWs **a** before and **b** after the O_2_ plasma treatment. **c** SiNWs after surface treatment and subjected to HF etching time of 85 s. A residual thin SiOx layer is seen on the SiNW surface

**Fig. 6:**
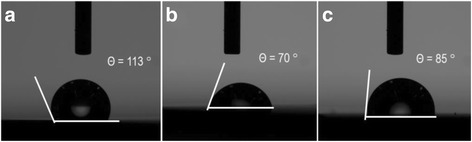
Contact angles of the SiNW substrate **a** as prepared, **b** after O_2_ plasma treatment, and **c** after HF etching

Figure 3 shows the top view and side view SEM images for two of the samples with 0.4- and 0.95-μm-long SiNWs coated with PEDOT:PSS, with and without surface treatment. It is observed that PEDOT:PSS forms a continuous canopy on top of the SiNWs instead of penetrating into the SiNW gaps, attributed to its long polymer chain [16]. Besides, while the shorter SiNWs are more vertically aligned, the longer SiNWs suffer from agglomeration at the top resulting in the formation of large bundles of SiNWs, ascribed to the van der Waals and attractive capillary forces [25, 26]. As a result, the PEDOT:PSS layer exhibits a bumpy surface contour on top of the SiNW bundles, and many SiNWs within the bundles are uncoated with PEDOT:PSS. In terms of the influence of surface treatment, it is noted that the treated SiNWs reveal a smoother surface coverage of PEDOT:PSS layer as compared to the untreated SiNWs. This could be attributed to the more hydrophilic surface of the SiNWs after the oxygen plasma treatment, which improves the PEDOT:PSS coverage in the spin coating process [27]. We have carried out transmission electron microscopy TEM studies of the treated SiNWs, as shown in Fig. 4, and similar to the results obtained for the two-step process using ozone treatment [11], it is observed that most of the Ag nanoparticles yielded during the MCEE process have been removed from the SiNW surface. Figure 5a, b shows the HRTEM images of a SiNW before and after the oxygen plasma treatment, respectively. As seen in Fig. 5a, the as-prepared SiNW has only a very thin SiOx layer on the surface due to its H+ terminated surface arising from the MCEE process. From Fig. 5b, it is seen that a thicker SiOx layer of 4 to 5 nm thickness was formed after the O_2_ plasma treatment. Figure 5c shows the SiNW after the 5 % HF etch for ~85 s, where a thin layer of 1 to 2 nm SiOx is seen to remain on the surface for passivation purpose. This SiO_x_ layer will change the wettability of the SiNW surface. Contact angle measurements have been carried out for the SiNW substrate before O_2_ plasma treatment, after O_2_ plasma treatment and after HF etching, the results of which are shown in Fig. 6a–c, respectively. It is seen that the two-step surface treatment changes the wettability of the SiNW surface from hydrophobic (contact angle *θ* = 113°) to hydrophilic (*θ* = 85°). The hydrophilic surface is advantageous as it promotes a smoother coverage of PEDOT:PSS on top of the SiNWs, as can be seen from the SEM images presented in Fig. 3.

**Fig. 7:**
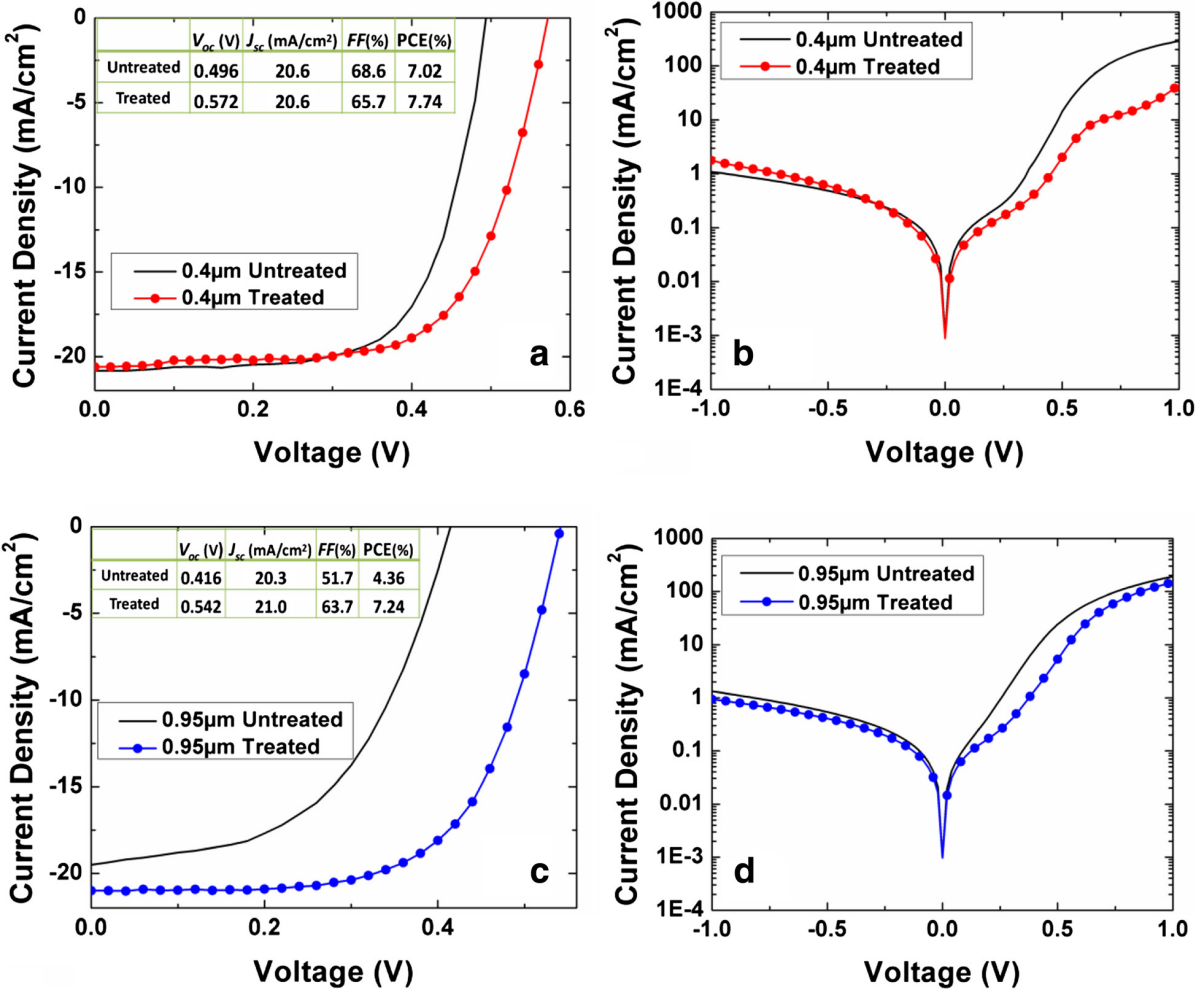
Current density-voltage (*J*-*V*) characteristics of 0.4-μm SiNW cells with and without surface treatment under **a** illumination and **b** dark condition. The corresponding results for 0.95-μm SiNW cells are shown in **c** and **d**, respectively

**Fig. 8:**
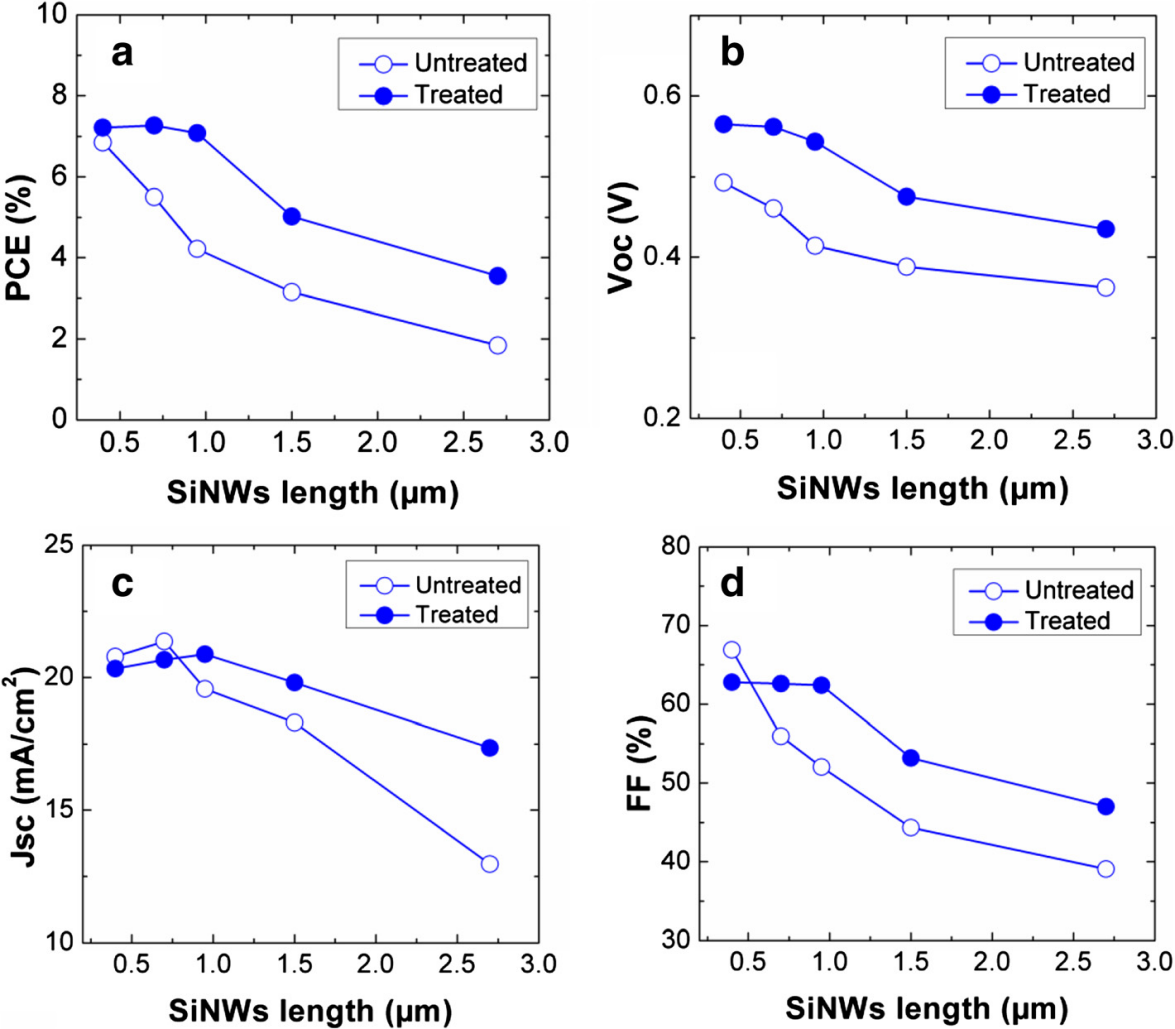
Average **a** PCE, **b** *V*_oc_, **c** *J*_sc_, and **d** *FF* for SiNW/PEDOT:PSS cells with and without surface treatment as a function of SiNW length

Four to six cells were fabricated and investigated for each length of the SiNWs. Figure 7a, b shows respectively the illuminated and dark *J-V* characteristics of a typical 0.4 μm SiNW/PEDOT:PSS hybrid cell without and with the two-step surface treatment. The corresponding results for the 0.95 μm SiNW/PEDOT:PSS hybrid cell are shown in Fig. 7c, d, respectively. Figure 8a–d summarizes the average photovoltaic parameters of the SiNW/PEDOT:PSS solar cells fabricated without and with the two-step surface treatment as a function of *L*. The illumination was carried out using a solar simulator (San-EI electric) under the AM 1.5G 100 mW/cm^2^ condition. The cells that are surface treated consistently exhibit better PCE as compared to the untreated cells of the same SiNW length. As seen in Fig. 8a, the average PCE exhibits a maximum value of 7.27 % at 0.7 μm, and it is less sensitive to the SiNW length, in contrast to the untreated cells where the PCE decreases sharply with increasing SiNW length. The better PCE is mainly due to the improvement in *V*_oc_ across all the samples, as well as higher *J*_sc_ and *FF* for samples with longer SiNWs. This is attributed to the surface treatment that resulted in the removal of impurity particles and defects on the SiNW surface, which is particularly crucial for longer SiNWs due to their large surface area. The *V*_oc_ of about 0.562 V measured for the shorter 0.7 μm SiNWs is much higher than those typically reported in the literature for untreated SiNW hybrid cells [6, 16]. Apart from the improved surface quality, the residual oxide layer resulted from the surface treatment also facilitates a favorable band alignment and leads to an internal electric field at the junction interface, leading to better charge separation [28–30]. In addition, the untreated cells exhibit lower shunt resistance, as evidenced from the early turn-on of the cells at low forward voltage range of the *J-V* curves seen in Fig. 7. This is ascribed to the rougher surface coverage of PEDOT:PSS layer on top of the SiNWs, which may lead to local shunting due to protrusion of the SiNW tips. This is expected to be more severe for longer SiNW cells, as the aggregation of SiNWs leads to a greater protrusion. In contrast, the treated cells have cleaner SiNW surface and better coverage of PEDOT:PSS layer, which also leads to the enhancement in carrier separation. The above factors account for the overall improved performance of the treated cells.

As seen in Fig. 8, the performance of the cells is poor at longer SiNW length, even for the treated cells. Due to the bundling, only SiNWs that are situated at the outer perimeter of the bundles are treated by the oxygen plasma, while those that are within the bundles are not well exposed to the plasma. Thus the surface treatment has limited effect on performance improvement for the very long SiNW cells, as compared to those with shorter SiNWs. Therefore, there is increased carrier recombination loss associated with the defective SiNW surface, which is compounded by the larger surface area of the longer SiNWs.

**Fig. 9:**
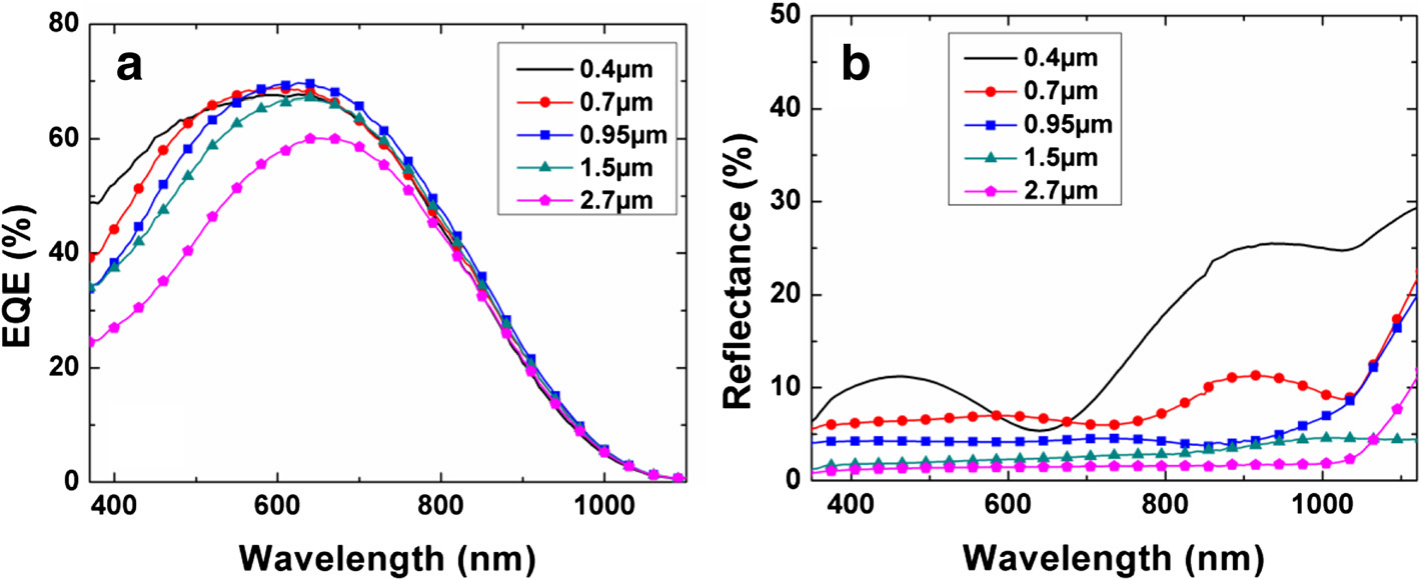
**a** *EQE* and **b** reflectance spectra of the SiNW/PEDOT:PSS solar cells as a function of SiNW length *L*

Figure 9a shows the *EQE* spectra of the SiNW/PEDOT:PSS cells with different *L*. The *EQE* peak increases with *L* up to *L* = 0.95 μm, with a maximum value of 69.7 % at *λ* = 625 nm. This is consistent with their reflectance spectra shown in Fig. 9b where the 0.95-μm SiNWs cell has a lower reflectance compared to the shorter SiNW cells. The high *EQE* is attributed to the enhanced light trapping and charge separation capability of the SiNWs, resulting in the highest *J*_sc_ of 20.88 mA/cm^2^, as shown in Fig. 8. As *L* increases to 1.5 and 2.7 μm, though the reflectance is further reduced to below 5 % owing to the more effective light trapping by the longer SiNWs, the *EQE* of the cells decrease monotonically, especially in the shorter wavelength range of 400–800 nm. This is indicative of higher recombination rate nearer the surface of the cells and is attributed to the formation of SiNW bundles, such that the SiNWs nearer the top are not properly treated and are also not well-coated with PEDOT:PSS to form junction. It is also noted that all the cells have lower EQE in the infrared region from 700 to 1100 nm as compared to similar cells based on bulk thick Si wafers [16]. This is because the thin epitaxial Si absorber layer does not fully capture longer wavelength photons. These photons are instead absorbed in the heavily doped Si substrate, where the carriers generated are not extracted due to the short minority-carrier diffusion length and high carrier recombination rate [8].

**Fig. 10:**
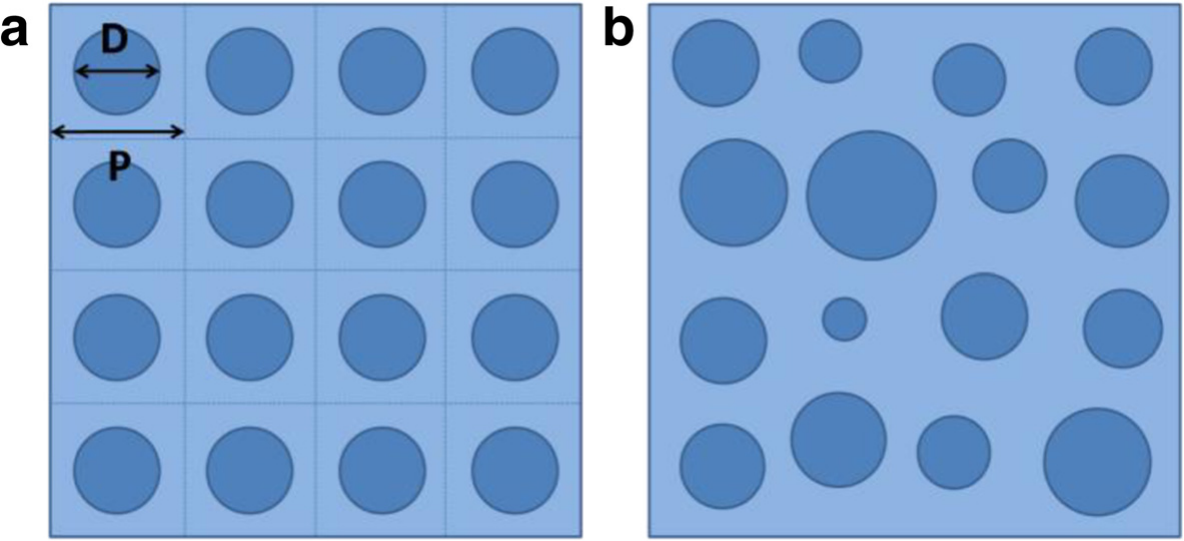
The top view of the schematic of the P-SiNW (periodic) and R-SiNW (random) structures simulated. D and P refer to the diameter and periodicity of SiNW in the P-SiNW, respectively

In this work, we also study the effect of the inherent randomness of the MCEE SiNWs on the light harvesting characteristics of the SiNW/PEDOT:PSS hybrid cell using the commercial software High Frequency Structural Simulator that is based on finite element method (FEM) [21]. The simulated cell structure consists of an array of SiNWs on a Si thin layer, with a total thickness of 2.2 μm, and it is coated with 50 nm thick of PEDOT:PSS on top. The SiNW length is fixed at 700 nm, which is the optimized length that we have deduced from our experimental studies. Figure 10 shows the top view of the periodic SiNW (P-SiNW) and random SiNW (R-SiNW) structures simulated. We observe experimentally that the MCEE SiNWs have typical diameter varying from 30 to 150 nm and the nearest spacing between adjacent SiNWs varying from 20 to 80 nm. Therefore, for the modeling of the P-SiNW, we assume a constant diameter (*D*) of 90 nm and spacing between adjacent SiNWs of 50 nm, which are the average of the values observed experimentally. Note that the simulated periodic structure has a periodicity of 140 nm. To model the R-SiNW, we consider a square unit cell with a side of 560 nm, which is four times the periodicity of the P-SiNW structure, so that it is equivalent in size to 16 unit cells of the P-SiNW structure. We assume 16 SiNWs distributed within the unit cell with the diameter and spacing randomly vary over the ranges of 30–150 nm and 20–80 nm, respectively, with a uniform random distribution. The random structural parameter values within the defined range are generated by Matlab and then fed into the optical simulation program. Periodic boundary condition is applied to the unit cell to simulate a large area two-dimensional structure. Plane wave over the range of 300 < *λ* < 1120 nm is incident normally onto the top surface of the hybrid structure. The electric field distribution resulting from the interaction of the incident sunlight and the nanostructure is solved to obtain the optical characteristics. The refractive indices of Si and PEDOT:PSS are obtained from literature [31, 32]. To ensure that the simulated optical characteristics of the R-SiNW structure sufficiently reflect the randomness of the SiNWs, the simulation process was repeated 12 times with different random parameters generated, and the results were then averaged. We have also confirmed that with about 12 simulations performed, there is already convergence of the average optical characteristics. Our approach reflects a quasi-random structure and helps to shed light on the absorption characteristics of the non-periodical hybrid structure.

**Fig. 11:**
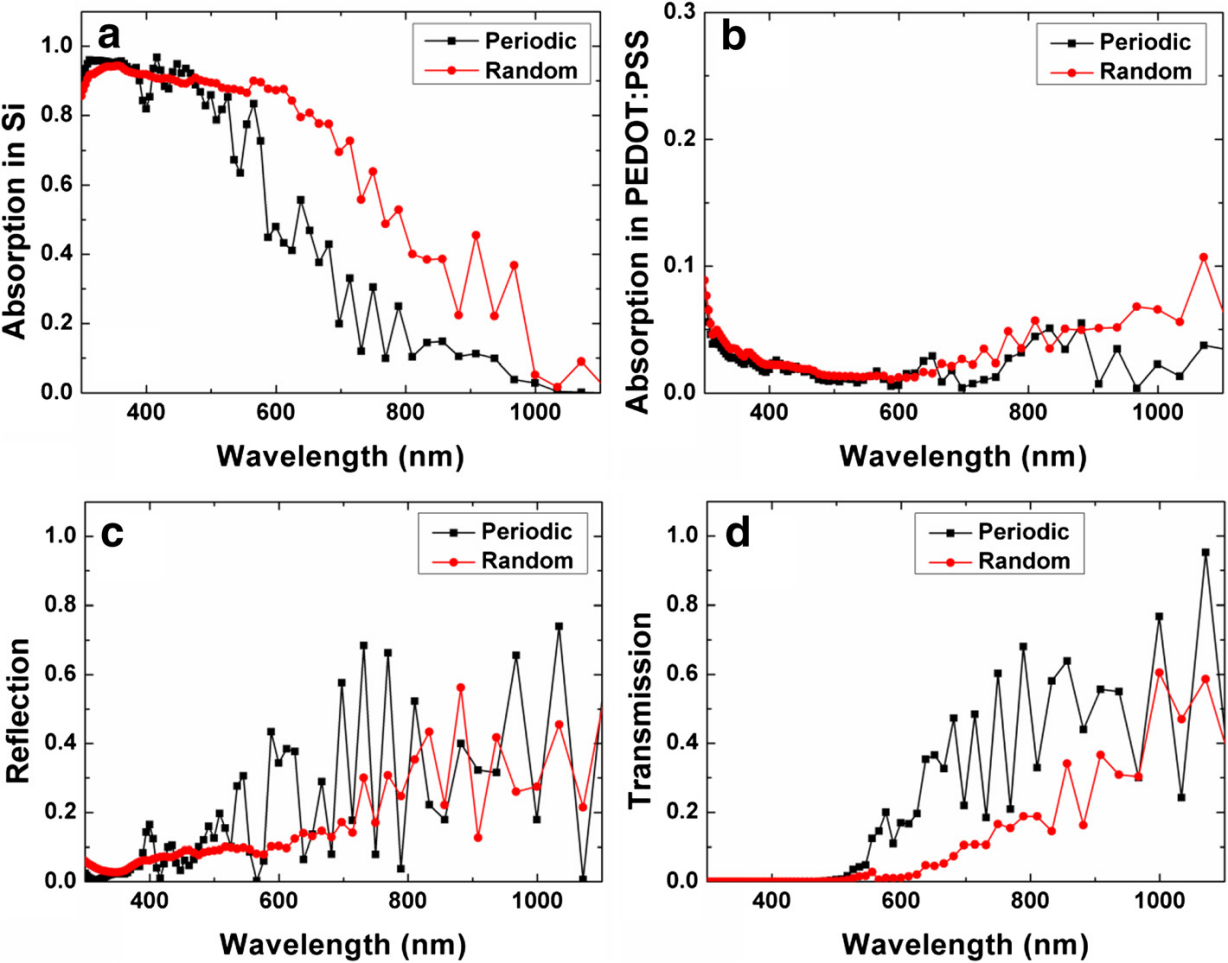
Simulated light absorption in the **a** Si layer and **b** PEDOT:PSS layer. **c** Reflectance and **d** transmission spectra of the P-SiNW (periodic) and R-SiNW (random) structures

The light absorption characteristics in the Si and PEDOT:PSS materials are shown in Fig. 11a, b respectively, whereas the overall reflectance and transmittance spectra of the hybrid structures are shown in Fig. 11c, d, respectively. It can be seen that the R-SiNW offers better light absorption than the P-SiNW, particularly at longer wavelengths where the smaller dimension of the P-SiNW with *P* = 140 nm does not lead to effective scattering of light [33]. It is also observed that the prominent resonant absorption peaks originally associated with P-SiNW are broadened in the case of the R-SiNW structure, and there is also presence of additional resonances. This is attributed to the fact that the structural symmetry is broken [34, 35] due to the varying diameter and spacing between the SiNWs. In addition, the R-SiNW structure would have some larger structural dimensions that enhance light scattering at longer wavelengths. The stronger trapping and scattering of light increases the optical path length and absorption and results in a low reflectance and transmittance for the R-SiNW, as can be seen in Fig. 11c, d, respectively. It is also noted that the light absorption in the PEDOT:PSS layer is stronger for the random structure, as there is stronger scattering of light at the longer wavelength range where PEDOT:PSS has relatively high absorption coefficients. However, this is not important as the absorption efficiency of Si in this wavelength range is very low. To quantify the effect of the randomness on the solar cell performance, we have compared the ultimate efficiency [36] of the P-SiNW and R-SiNW structures and found that it is substantially improved from 16.9 to 27.2 % when there is randomness in the SiNW array.

According to the simulation results, randomness in the SiNW structure is beneficial and it accounts for the excellent light harvesting ability of the MCEE SiNWs, in spite that their structural dimensions are much smaller than the wavelengths of light in the main solar spectrum. It is noted that the simulated reflectance spectrum of the R-SiNW structure is not as low compared to the experimental results shown in Fig. 9b, especially in a longer wavelength range. The deviation can be attributed to the fact that our simulation does not reflect a truly random structure but rather a quasi-random one. Besides, there are other factors not taken into account in our simulation, which include variation in the SiNW length, non-vertical alignment of the SiNWs, the rough surface and the imperfect cylinder shape of the SiNWs, and the rough coverage of the PEDOT:PSS layer on SiNWs. In addition, due to the computing resource constraints, our simulation was performed for a thinner Si film of 2.2 μm instead of the thicker 10.6 μm Si film that was used in the experimental structure. Nevertheless, the simulation results clearly reveal the effect of the randomness of the MCEE SiNWs on enhancing the scattering and absorption of light in SiNW/PEDOT:PSS hybrid cells.

Our experimental cell structure is not optimized due to the absence of a back reflector. There is also parasitic optical loss in the PEDOT:PSS as light is incident on the top PEDOT:PSS layer. If these issues are addressed, it is expected that the performance of the thin film cells will be substantially improved. Indeed, recently, a high PCE of 10.3 % has been reported for planar multicrystalline Si/PEDOT:PSS solar cell based on the backPEDOT structure, using a Si absorber thickness of 5 μm passivated with Al_2_O_3_ [22]. The performance of such thin film-based Si/PEDOT:PSS cells can be further improved by enhancing optical absorption in the long wavelength range, through having a good light trapping scheme such as the one demonstrated in this work based on SiNWs with the surface treatment. The promising performance, coupled with the simple cell structure fabricated using low cost and low temperature process, will render such thin film Si/PEDOT:PSS hybrid structure attractive for low cost and high efficiency solar cells applications.

## Conclusions

We have demonstrated thin film SiNW/PEDOT:PSS solar cells using a 10.6 μm Si absorber. High efficiency of 7.83 % has been achieved for 0.7-μm-long SiNW cells with surface treatment. The light harvesting ability of the MECC SiNWs is studied both experimentally and theoretically. The inherent randomness of the low cost MCEE SiNWs is found to be beneficial for light trapping and absorption in the hybrid solar cell. The promising results obtained demonstrate the potential of realizing efficient Si/PEDOT:PSS hybrid solar cells using thin film Si.
